# *i*Drug: a web-accessible and interactive drug discovery and design platform

**DOI:** 10.1186/1758-2946-6-28

**Published:** 2014-05-23

**Authors:** Xia Wang, Haipeng Chen, Feng Yang, Jiayu Gong, Shiliang Li, Jianfeng Pei, Xiaofeng Liu, Hualiang Jiang, Luhua Lai, Honglin Li

**Affiliations:** 1 Shanghai Key Laboratory of New Drug Design, State Key Laboratory of Bioreactor Engineering, School of Pharmacy, East China University of Science and Technology, Shanghai 200237, China; 2 School of Information Science and Engineering, East China University of Science and Technology, Shanghai 200237, China; 3 BNLMS, Center for Quantitative Biology, State Key Laboratory for Structural Chemistry of Unstable and Stable Species, College of Chemistry and Molecular Engineering, Peking University, Beijing 100871, China

**Keywords:** Online drug design platform, Cavity detection, Pharmacophore search, 3D similarity calculation, Target prediction

## Abstract

**Background:**

The progress in computer-aided drug design (CADD) approaches over the past decades accelerated the early-stage pharmaceutical research. Many powerful standalone tools for CADD have been developed in academia. As programs are developed by various research groups, a consistent user-friendly online graphical working environment, combining computational techniques such as pharmacophore mapping, similarity calculation, scoring, and target identification is needed.

**Results:**

We presented a versatile, user-friendly, and efficient online tool for computer-aided drug design based on pharmacophore and 3D molecular similarity searching. The web interface enables binding sites detection, virtual screening hits identification, and drug targets prediction in an interactive manner through a seamless interface to all adapted packages (e.g., Cavity, PocketV.2, PharmMapper, SHAFTS). Several commercially available compound databases for hit identification and a well-annotated pharmacophore database for drug targets prediction were integrated in *i*Drug as well. The web interface provides tools for real-time molecular building/editing, converting, displaying, and analyzing. All the customized configurations of the functional modules can be accessed through featured session files provided, which can be saved to the local disk and uploaded to resume or update the history work.

**Conclusions:**

*iD*rug is easy to use, and provides a novel, fast and reliable tool for conducting drug design experiments. By using *i*Drug, various molecular design processing tasks can be submitted and visualized simply in one browser without installing locally any standalone modeling softwares. *i*Drug is accessible free of charge at http://lilab.ecust.edu.cn/idrug.

## Background

Computer-aided drug design (CADD) is a widely used term that represents computational tools and sources for the storage, management, analysis and modeling of compounds [1]. Benefiting from the dramatic increase of biomacromolecular and small molecular information, CADD techniques are used at various stages of a drug-discovery project, from target identification and validation to lead discovery and optimization, even extending to preclinical trials [2].

A huge body of CADD software has been developed over the years in many different research groups [3-11]. However, most molecular design packages, with single client interface and powerful algorithms, may require separate license keys to be purchased individually. In addition, due to the complexity and diversity of molecular design procedures, sometimes even trained computational chemists can not perfectly excel in what they are computing [12]. In parallel, the open source and easily accessible software, which provides a great opportunity to perform research in CADD, has often encountered two main problems when being used by medicinal chemists [13]. First, in many instances the authors of CADD software pay more attention to scientific details rather than the usability of software, thus making it rarely associated with the graphical user interface and difficult to use for non-expert experimentalists. Second, individual program usually requires a specific input structure and produces a specific output format, making users incapable of combining different programs.

The striking growth of web technologies provides an alternative strategy to offer the possibility of accessing and running computational chemistry tools directly on the web with a simplified user interface. The major advantages of these web-based solutions are that users can submit models and data to the online servers without regularly downloading and updating the data collections and tools to their local disks. An increasing number of web applications for performing molecular modeling and processing are also available for end users [14-17]. However, these tools are always not able to integrate various molecular design programs and enormous data collections to meet the systematic operations of users to achieve the best results [18]. Besides, even if some initiatives combining different computational programs exists, e.g. for e-LEA3D [19], Sanjeevini [20], only focused on structure-based drug design such as molecular docking and de novo drug design which rely on the knowledge of the given structure of the target macromolecule.

**Figure 1:**
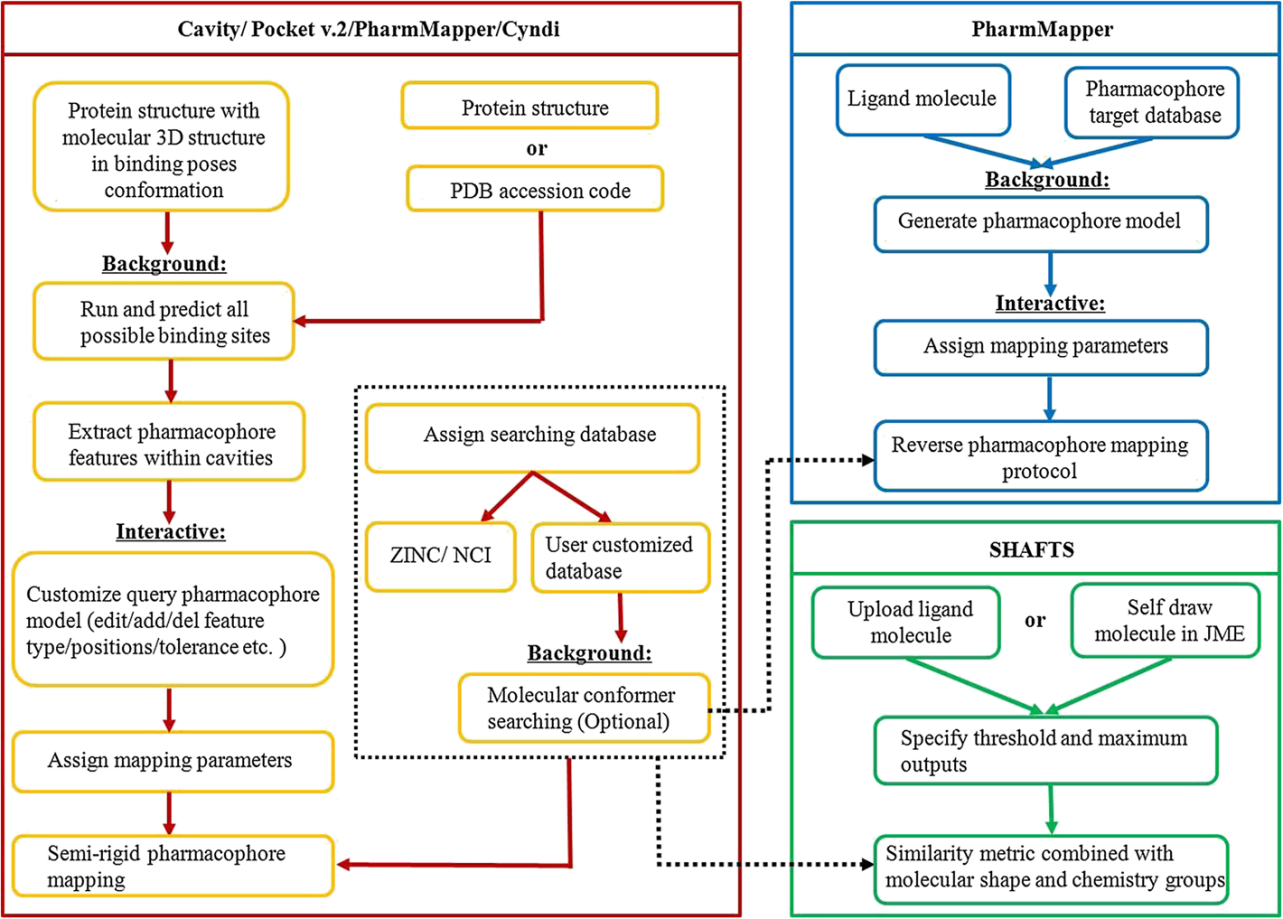
**Workflow of *i*Drug automating pharmacophore modeling using Cavity and Pocket v.2, screening with PharmMapper and SHAFTS, and searching conformers using Cyndi.** Common modules in *i*Drug platform are framed in black.

Here, we developed an online interactive platform, termed *i*Drug, to break expensive commercial suites and command line barrier and introduce a user-friendly web environment to conduct 3D molecular similarity calculation and to construct pharmacophore models for virtual screening. The user may select the molecular processing engine used by *i*Drug and setup and initiate jobs. Currently the system allows access to derive pharmacophore models directly from the given receptor regardless of a molecular 3D structure in the corresponding binding pose conformation provided as the reference (Cavity [8] and PocketV2 [21]); generate novel molecules for the given site using pharmacophore mapping approach; predict targets of a chemical of interest such as drugs, lead compounds and natural products (PharmMapper [22]); and rank candidates based on similarity-based database searching (SHAFTS [7,23,24]). Different modules described above (Table 1) have been incorporated, which work in a pipeline as depicted in the architecture (Figure 1). It also features a session based working bench to save, resume, and reuse the jobs and configurations customized by users, which can be accessed and updated through the interface easily. As *i*Drug addresses common problems associated with either biomacromolecule or small molecule, it is expected to help both experts and non-specialists to achieve the automated molecular design of daily research demands, as well as being a routine adjunct to experimental studies.

**Table 1:** **List of computational techniques supported by *i*** **Drug**

**Name**	**Method**	**Refs**	**Free for academia**
Cavtiy	Detect and score potential binding sites of a protein	[8,33]	Yes
Pocket v.2	Derive pharmacophore models based on a given receptor of complex structure	[21]	Yes
PharmMapper	Pharmacophore mapping (online web service)	[22]	Yes
SHAFTS	3D similarity calculation	[7,23]	Yes
Cyndi	Molecular conformation generation	[38,39]	Yes
Pybel	Python wrapper for the OpenBabel cheminformatics toolkit	[25,26]	Yes

## Methods

### Implementation and interface

The *i*Drug system was developed using Java on Apache Tomcat Server. The platform back-end was developed in Python programming language, with MySQL as the database system, while the front-end is implemented with JSP using JavaScript and AJAX technologies utilizing the jQuery library. The open-source Java viewer Jmol (http://www.jmol.org/) is used for 3D display and manipulation of molecular structures. Pybel [25,26], a Python wrapper of the OpenBabel toolkit, was used in backend for molecular file parsing and converting.

**Figure 2:**
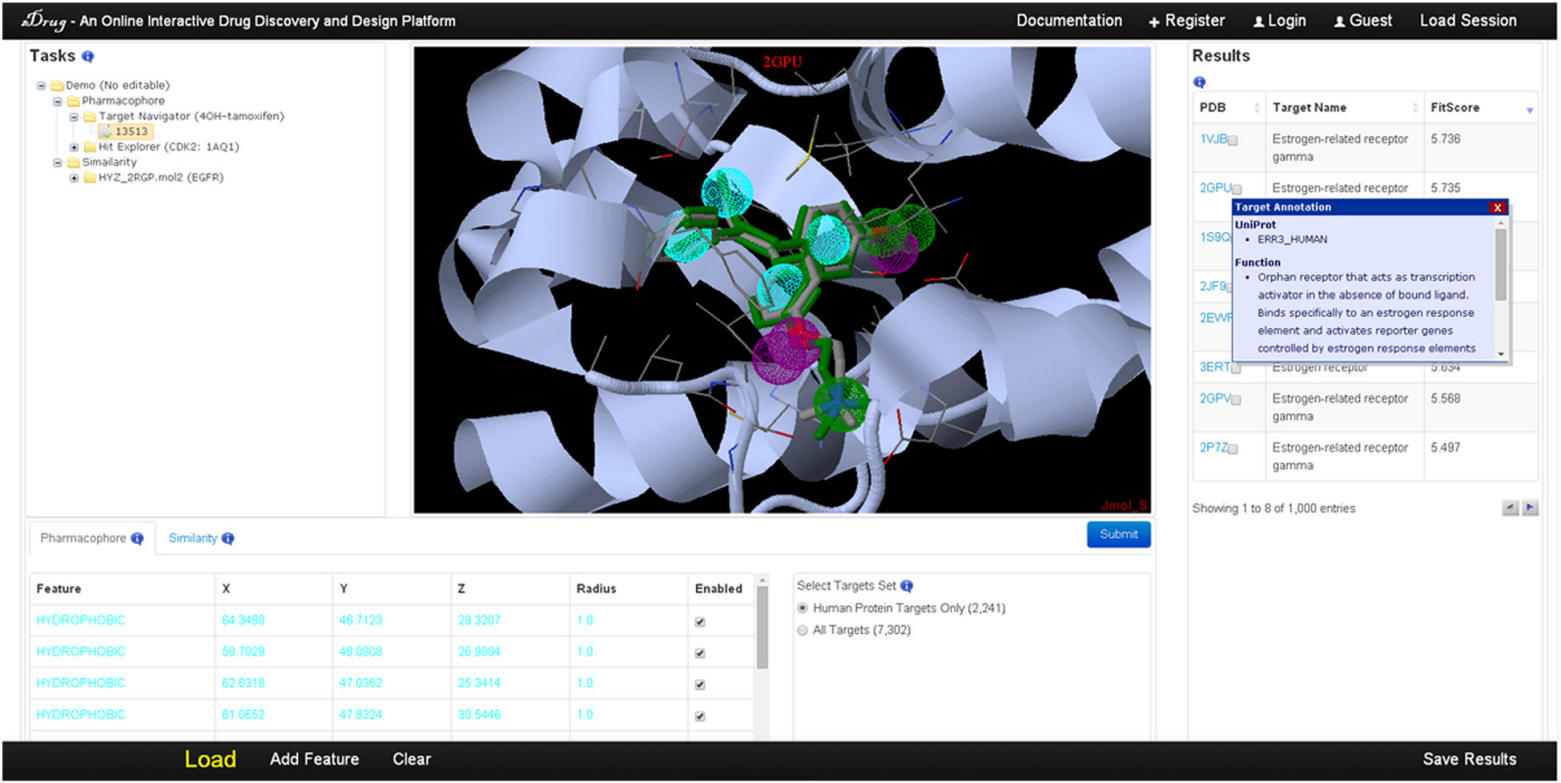
**The *i*Drug interface.** The task management is in the upper left and provides easy access to the full set of the history work. The Jmol-based molecular viewer is in the middle and displays the query molecule and results structure. The query editor is shown in the bottom and supports the interactive modification of the parameters based on the properties of the computational software. The results browser is on the right and displays the complete results along with the available details. In this figure, potential targets of tamoxifen obtained from *i*Drug are shown as well as the target pharmacophore model. On mouse over, a preview of the annotation information is displayed in a pop-up window, as shown for 2GPU in this example.

The user interface of *i*Drug is shown in Figure 2. Users can normally start a session by clicking the ‘Load’ button. As *i*Drug provides pharmacophore- and similarity-based tasks, the corresponding uploading dialog is grouped into two modes: ‘pharmacophore work mode’ and ‘similarity work mode’. After a successful submission, a unique Job ID is assigned and used to access the computational results. Result files associated with the completed jobs are stored at the server for 3 months, which can be downloaded for offline analysis within this period of time.

### Chemical and drug target database

*i*Drug currently integrates the publicly accessible NCI database [27] and the commercially available and biological relevant compounds from ZINC [28], comprising over 3 million compounds with multiple 3D conformers pre-generated to facilitate both pharmacophore and 3D similarity based virtual screening. An in-house pharmacophore database called PharmTargetDB [22] containing over 7,000 pharmacophore models derived from complex crystal structures with corresponding protein target annotations is incorporated in *i*Drug as well. The target protein annotations including name, function, and involved indication/disease were referred from DrugBank [29], BindingDB [30], PDBBind [31], and PDTD [32] databases. The key information for each database was summarized in Table 2.

**Table 2:** **Data source of *i*** **Drug**

**Type**	**Database**	**Number of entities**
**Compounds**	ZINC Lead-like	3,027,615
	NCI Open Database	246,483
**Targets**	PharmTargetDB	7,302

### Cavity detection and structure-based pharmacophore modeling

Identification of protein cavities is of fundamental importance for structure-based pharmacophore derivation. *i*Drug applies Cavity to detect the potential binding sites on the surface of a given protein structure, and rank them according to the corresponding druggability scores on the basis of geometric shape, hydrogen bonding, and hydrophobic effects. By comparison of the success rate for the first-ranked prediction tested on the PDBBind dataset, Cavity outperformed four popularly used binding sites detection approaches (LIGSITE*csc*, Q-sitefinder, SURFNET, and PASS), indicating its accurate prediction of relatively small binding sites on the surface of a protein [8,33]. Li *et al*. customized LigBuilder 2.0 to generate and fit the shape of the Cyclophilin A (CypA) binding site and finally obtained two highly potent inhibitors with nanomolar inhibitory potencies (2.59 nM and 1.52 nM) [34]. A receptor-based pharmacophore modeling program Pocket v.2 was used to extract pharmacophore features within cavities [21]. In this approach, hotspot analysis and grid-based scoring were used to identify essential pharmacophore features. As suggested by case study results, Pocket v.2 can yield consistent pharmacophore models for proteins with minor conformational changes upon binding of different ligands, indicating the robustness of the approach. Lai *et al.* proposed a novel strategy that combined receptor-based common pharmacophores with molecular docking. Three compounds were identified through this strategy to inhibit LTA4H-h and hnps-PLA2 simultaneously [35].

Normally, *i*Drug requires a protein 3D structure or a known PDB accession code as input to derive pharmacophore models. All binding sites along with the extracted pharmacophore features will be displayed and ranked by the druggability scores. A small molecular 3D structure in the corresponding binding poses conformation can also be uploaded as the reference to specify the position of the binding site and generate the pharmacophore features representing the corresponding interaction mode. It takes averagely 1–3 minutes for binding sites detection, and the potential binding sites can be interactively viewed and downloaded in PDB files for off-line analysis, which includes the binding pocket surface, the residues around the site, and key features of the pharmacophore model derived from the site.

### Pharmacophore mapping and virtual screening

For pharmacophore-based screening, *i*Drug employs a pharmacophore mapping method called PharmMapper based on feature triplet hashing and searching algorithm [22]. Liu *et al.* discovered two potent IGF-1R kinase inhibitors via hierarchical strategy based virtual screening (pharmacophore screening and docking), which efficiently reduced the number of “nonhits” passed to docking stage and consequently reduced the computational cost [36].

The query pharmacophore model can be derived from the potential binding sites detected previously or user customized ones. *i*Drug allows interactive editing of the features of the pharmacophore query like feature type, positions, and size of the tolerance spheres. Pharmacophore searching is performed against the available or user uploaded databases. The hit compounds are compiled into the table with the corresponding original weblinks whereas applicable, and ranked by the fit values with the query. The superimposed mode of the molecules onto the pharmacophore query along with the corresponding binding site can be interactively visualized in Jmol applet.

### Target identification

*i*Drug uses the reverse pharmacophore mapping procedure to predict potential drug targets. Qian *et al.* discovered a novel series of acenaphtho[1,2-*b*]pyrrole derivatives as potent FGFR1 inhibitors by using this in silico targets screening approach [37]. The results were subsequently validated by enzyme-linked immunosorbent assay. The platform takes a single drug-like molecule or natural product chemical structure as an input and automatically perceives all chemotype features of the query molecule. Users are allowed to modify the perceived pharmacophore model and search the pharmacophore target database with it. The results can also be filtered in terms of both the number of returned results and the properties of the algorithm. Since the geometric matching alignment and scoring method uses rigid conformations, multiple conformers of each query molecule must be generated. In-house program Cyndi [38,39], which uses a multi-objective evolution algorithm method for conformer searching, was chosen by default to generate multiple conformation. Additionally, the minimum number of each pharmacophore feature type and a fit score cutoff can be specified to discard those target pharmacophore models, of which the corresponding values are less than the threshold. On average, the total time consumed by the complete screening and scoring protocol ranges from 1-2 h depending on the flexibility of the input molecule and filter parameters assigned by the user.

Upon completion of the computations, the results of the hit target pharmacophore models are demonstrated in the form of a ranked list. Each row contains the protein ID, which is a hyperlink points to the PDB website [40], the target name, the fit values between the small molecule and the pharmacophores, and a unique orientation of a conformation of the query along with the 3D structure information of the target visualized by the Jmol applet. Detailed annotations of the hit targets are presented in a pop-up window when the cursor moves over the corresponding PDB codes. Alternatively, a downloadable zip file containing the aligned pose with the corresponding pharmacophore model and the complete targets information in CSV format is accessible for each returned match.

### Similarity search

For the implementation of ligand-based searching, *i*Drug adopts an efficient 3D similarity calculation method SHAFTS, which is designed to integrate the strength of pharmacophore matching and volumetric overlay approaches [7]. Hits are determined by a hybrid similarity score cut-off and alignment poses of compounds will be generated by enumerating all potential pharmacophore feature triplets matches. Liu *et al.* discovered sixteen compounds with IC_50_ < 20 μM, three of which showed low micromolar inhibitory activities against p90 ribosomal S6 protein kinase 2 (RSK2) and exhibited selectivity across a panel of related kinases using SHAFTS [23]. By adopting the same strategy, Xu *et al.* reported a novel pteridin-7(8*H*)-one epidermal growth factor receptor (EGFR) inhibitor scaffold with potent and selective inhibitory activity against both wild-type and T790M/L858R mutant EGFRs. The most remarkable agent showed highly inhibitory activity against the growth of gefitinib-resistant H1975 cells, making it a potential lead for further development of EGFR kinase-related anticancer drugs [41]. The results suggests that SHAFTS is an efficient and powerful tool in scaffold hopping and hit identification endeavors. Moreover, Shen *et al.* used the evodiamine derivative as a probe to search MDL/Symyx Drug Data Report (MDDR) [42] with ChemMapper. The fourth ranked protein, topoisomerase II (Top2), was a well-known antitumor target. Relaxation assay proved that the analogs of the natural product are potent inhibitor against Top2, with stronger activity than the well-known Top2α inhibitor etoposide [43].

A chemical structure (sketched online or uploaded in multiple chemical structure file formats) is accepted as the query to perform similarity searching. It takes hours to days per search depending on the complexity of the query molecule and the size of the library.

The outputs are generated as a list of compounds sorted by similarity scores to the query and can be downloaded in a mol2 file. The superimposed pose of each hit with the query can be visualized interactively in Jmol applet along with the molecular surface representation and perceived pharmacophore features by SHAFTS.

## Results and discussion

### Benchmarking study

To demonstrate potential applications of *i*Drug platform, we performed pharmacophore-based virtual screening libraries with the MUV data sets [44], molecular 3D similarity-based virtual screening with the enhanced Directory of Useful Decoys (DUD-E) [45] data sets, and reversed pharmacophore mapping-based drug target identification with the pharmacophore target database. Receiver Operator Characteristic (ROC) curves, Area Under the ROC Curves (AUC), and enrichment factors (EF) were calculated after ranking compounds from the MUV and DUD-E data sets. EF after x% of the library screened were calculated according to the following formula (*Hits*_*sampled*_ = number of hits found at x% of the database screened, *N*_*sampled*_ = number of compounds screened of x% of the database, *N*_*total*_ = the number of compounds in the entire database, *Hits*_*total*_ = the number of actives in the entire database).

(1) $$\mathrm{EF}=\frac{\mathrm{Hit}s_{\mathrm{Sampled}}}{N_{\mathrm{Sampled}}}\times \frac{N_{\mathrm{Total}}}{\mathrm{Hit}s_{\mathrm{Total}}}$$

### Case 1: pharmacophore-based virtual screening

**Figure 3:**
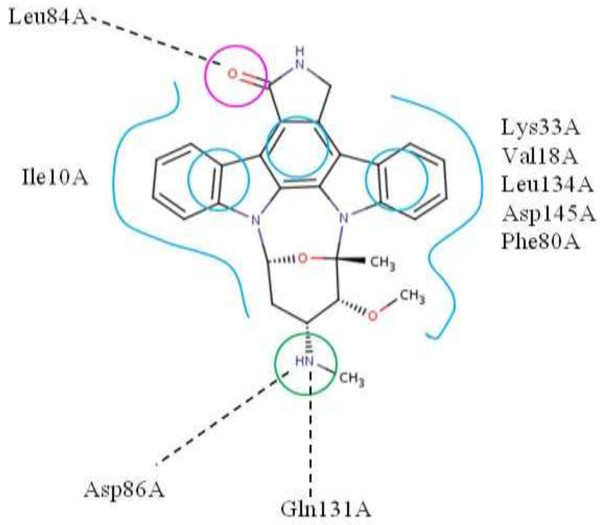
Pharmacophore depiction as used in this study on top of PDB entry: 1AQ1 (note that 1AQ1 with its cocrystallized ligand is used as a reference).

CDK2 (Cyclin-dependent kinase 2) is a protein kinase whose pharmacophore features, depicting ligands that target against the ATP binding site, are well described in the literature [46]. We used *i*Drug to create pharmacophore queries from the crystal structures of CDK2 (PDB:1AQ1). The pharmacophore queries occupied by the bioactive conformation of the ligand, which contain one H-bond acceptor, one H-bond donor and three hydrophobic features, were selected as the hypotheses (Figure 3).

We create the compound sets using the virtual screening dataset of CDK2 comprising 80 active compounds and 15000 decoy compounds [47]. The search of 249,242 conformers of 15080 compounds takes almost 20 minutes. Without any prescreening, *i*Drug matches 50 out of 80 actives and 5641 out of 15000 decoys resulting in an enrichment factor of 1.7. The AUC value is 0.63, indicating that the overall enrichment is only slightly better than that expected from a random selection (Table 3). This is an important observation suggesting that though not so effective in actives enrichment with single pharmacophore model, *i*Drug enables fast prefiltering for large compound collections before applying more accurate and computationally expensive algorithms.

**Table 3:** AUC value and EF values at 0.5, 1, 2 and 5% for CDK2 inhibitor pharmacophore-based virtual screening

**AUC**	**EF**			
0.63	0.5%	1.0%	2.0%	5.0%
	2.5	1.3	2.5	2

### Case 2: target prediction

Tamoxifen, which is used as an adjuvant therapy in the treatment of breast cancer, has been proved as a multiple target drug. So far, tamoxifen and its active metabolite, 4OH-tamoxifen, are known to interact with 16 targets. We have chosen to investigate 4OH-tamoxifen as a test case for validation (the typical run takes less than an hour). The 11 known targets of 4OH-tamoxifen retrieved by *i*Drug are shown in Table 4. Other targets, including microsomal antiestrogen binding site (AEBS) [48], cholesterol acyl transferase (ACAT) [48], cholesterol epoxide hydrolase [49], hedgehog signaling [50], immunoglobulin [51], are missed due to the limited coverage of the pharmacophore database. In spite of this, the reverse pharmacophore mapping approach, therefore, enables 11 of 16 experimentally confirmed tamoxifen targets to be retrieved within 10% of the ranked database, which is promising and reliable for a retrospective target identification case.

**Table 4:** **Retrieval of 11 targets of 4OH-Tamoxifen by *i*** **Drug**

**Target name**	**Reference**	**Score**	**PDB ID**	**Rank(%)**
Estrogen receptor-γ	[52]	5.736	1VJB	0.01
Estradiol 17β-hydroxysteroid dehydrogenase 1	[53]	4.111	1I5R	0.26
Dihydrofolate reductase	[54]	3.777	1DG7	0.45
Glutathione S-transferase A1	[55]	3.655	1GSF	0.73
Prostaglandin G/H synthase 2	[56]	3.411	1PXX	1.36
Liver carboxylesterase 1	[57]	3.344	1YA4	1.59
Protein kinase C theta type	[58]	3.171	1XJD	2.18
Calmodulin	[59]	2.974	1XA5	3.63
Collagenase 3	[60]	2.945	3I7I	4.57
Alcohol dehydrogenase E chain	[60]	2.881	1MGO	7.37
3-alpha-(or20-beta)-hydroxysteroid dehydrogenase	[61]	2.835	1HDC	9.87

### Case 3: molecular 3D similarity-based screening

The crystal ligand structure of epidermal growth factor receptor (EGFR, PDB:2RGP) was searched via *i*Drug. The screening library was created using the DUD-E data sets including 542 active compounds and 35,050 decoy compounds for a baseline enrichment of 1.5%. A multiconformer library was generated using Cyndi with the default settings (up to 200 conformers per a compound), resulting in a library of 2,735,015 conformations.

The search of EGFR query in *i*Drug takes 12–14 hours. The performance of *i*Drug with respect to compound library enrichment, i.e., the fraction of true positives versus the fraction of false positives, was visualized in ROC. The corresponding AUC and enrichment values at 0.5, 1.0, 2.0, and 5.0% are calculated and reported, which can be found in Table 5. The AUC value is 0.87 and the EF value at the 0.5% level is 56.9, indicating highly significant enrichment with respect to random ones.

**Table 5:** AUC value and EF values at 0.5, 1, 2 and 5% for EGFR inhibitor similarity virtual screening

**AUC**	**EF**			
0.87	0.5%	1.0%	2.0%	5.0%
	56.9	42.9	28.5	13.6

## Experimental

### Compound data sets

Datasets from four different public sources were used. A set of over 3 million compounds extracted from ZINC [28] and NCI [27] were used for both pharmacophore and 3D similarity based virtual screening. Cyndi [38] was used to generate a maximum of 50 low-energy conformations for each compound. The MUV data sets are based on bioactivity data. MUV consists of 17 targets, each with 30 actives and 15000 decopys [44]. In this study we reproduced the evaluation of Sanders *et al*[47] and derived pharmacophore queries from the crystal structure of CDK2 (PDB:1AQ1). The DUD-E was originally designed for benchmarking of docking methods [45]. A subset was later extracted for the use in 3D similarity-based virutal screening experiments. The DUD-E contains 102 targets with 22886 clustered ligands drawn from ChEMBL, each with 50 property-matched decoys drawn from ZINC. In this study EGFR with 542 actives were used. Besides, for the user uploaded target compounds, multi conformations (at most 100) would be generated online with Cyndi prior to virtual screening if the ‘Generate Conformers’ is toggled on.

### Target pharmacophore databases

The target protein structures co-complexed with small molecules were selected from DrugBank, BindingDB, PDBBind, and PDTD databases. All the small ligands with molecular weight lower than 100, such as solvents, buffers and metal cations, and all the cofactors with molecular weight over 600, such as CoAs, polypeptides and nucleic acids were regarded. For the proteins existing as homopolymers, only one monomer was reserved for analysis. For the proteins determined by NMR with multiple structure models, only the first model was selected for pharmacophore generation. LigandScout [9] was used in the process of pharmacophore model derivation. As a result, a database with 7302 pharmacophore models were generated and stored in *i*Drug. This database has been used in our previous publications [22].

## Conclusions

We presented *i*Drug, a versatile web server for both pharmacophore- and similarity-based virtual screening and target identification to facilitate computational drug discovery. The interface is easy-to-use and can be accessed by user customized sessions to free them from installing standalone softwares. *i*Drug provides ready-to-access compounds and pharmacophore target databases for virtual screening and target identification. Various applications like binding site identification, structure-based pharmacophore derivation, conformational sampling, pharmacophore searching, and 3D similarity calculation are integrated as individual modules. It enables interactive editing and refinement of pharmacophore hypothesis as well as flexible customization of the parameters. The featured job management system ensures the user privacy and project tracking through a session-based mechanism.

## Competing interests

The authors declare that they have no competing interests.

## Authors’ contributions

HL designed the project. XW analyzed the data, contributed the use cases and wrote the manuscript. HC and FY supported the project with background and front-end development. JG contributed the back-end schedule system. JP, XL, HJ, LL, and HL contributed the molecular design approaches. All authors read and approved the final manuscript.
